# COVID-19 Infection, the COVID-19 Pandemic, and Changes in Sleep

**DOI:** 10.3389/fpubh.2021.795320

**Published:** 2022-01-31

**Authors:** Sidney M. Donzella, Lindsay N. Kohler, Tracy E. Crane, Elizabeth T. Jacobs, Kacey C. Ernst, Melanie L. Bell, Collin J. Catalfamo, Rachelle Begay, Kristen Pogreba-Brown, Leslie V. Farland

**Affiliations:** ^1^Department of Epidemiology and Biostatistics, University of Arizona, Tucson, AZ, United States; ^2^Department of Epidemiology, University of Washington, Seattle, WA, United States; ^3^University of Arizona Cancer Center, Tucson, AZ, United States; ^4^Department of Health Promotion Science, University of Arizona, Tucson, AZ, United States; ^5^Department of Medical Oncology, University of Miami, Miami, FL, United States

**Keywords:** sleep, sleep quality, SARS-CoV-2 infection, COVID-19 pandemic, sleep patterns

## Abstract

The objective of this study was to investigate the differences in sleep patterns among individuals with and without laboratory-confirmed SARS-CoV-2 infection. Laboratory-confirmed SARS-CoV-2 test results and self-reported measures recalling sleep habits prior to and during the pandemic were collected from May 2020 to March 2021 among 1,848 individuals in The Arizona CoVHORT Study. We used linear and logistic regression to model the association between test status, presentation of symptoms, and time since test result with sleep duration and trouble sleeping, respectively. Mixed models were used to investigate change in sleep duration prior to the pandemic compared to during the pandemic. Overall, 16.2% of the sample were SARS-CoV-2 positive, 64.3% were SARS-CoV-2 negative, and 19.5% were untested for SARS-CoV-2. Independent of SARS-CoV-2 infection status, all participants slept longer during the pandemic compared to pre-pandemic (Δ SARS-CoV-2 positive: 77.7 min, 95% CI 67.9, 87.5; Δ SARS-CoV-2 negative: 13.4 min, 95% CI 8.4, 18.3). However, SARS-CoV-2 positive participants slept 60.9 min longer (95% CI 49.1, 72.8) than SARS-CoV-2 negative participants in multivariable-adjusted models and had greater odds of trouble sleeping three or more times per week since the start of the pandemic (OR: 1.34 95% CI 1.02, 1.77) This greater odds of trouble sleeping persisted for participants who reported sleep habits > 30 days after their positive SARS-CoV-2 (OR: 2.11 95% CI 1.47, 3.03). Sleep patterns among non-hospitalized individuals with COVID-19 were altered following infection, regardless of the presentation of symptoms and time since infection.

## Introduction

Since October 2021, the Centers for Disease Control and Prevention has reported more than 43 million confirmed cases of COVID-19 in the United States (1). When evidence indicated asymptomatic and pre-symptomatic transmission contributed substantially to the growing pandemic (2), lockdown measures were implemented to enforce physical and social distancing. By May 31, 2020, 43 US states and territories had issued shelter-in-place (or stay-at-home) orders (3). For many, the unprecedented challenges arising from the COVID-19 pandemic, including infection with SARS-CoV-2, have disrupted many lifestyle habits that may influence sleep-wake cycles.

Sleep is critical to overall health across the lifespan. Studies have shown the bidirectional association of sleep duration and sleep quality with physical and psychological health (4–6). Timing of the sleep-wake cycle is associated with health outcomes (7) and researchers have found later bedtimes to be associated with worse physical health (8). Sleep habits are driven by social, environmental, and societal factors (9). Previous research has shown that the COVID-19 pandemic has altered sleep habits among the general population and healthcare workers (10, 11). More specifically, it has been reported that sleep quality has worsened and the sleep phase has shifted later (10, 12, 13).

As the number of COVID-19 cases continues to increase, understanding how infection with the SARS-CoV-2 virus may influence sleep is imperative. Previous research has highlighted broadly the potential changes in sleep induced by the immune response post-infection (14). Laboratory and observational studies demonstrate that after infection with viruses that result in common cold- and flu-like illnesses, sleep duration, sleep quality, and sleep onset can vary depending on presentation of symptoms (15, 16) and time since infection (16, 17). These alterations in sleep may depend on the specific pathogen, host, and route of infection (14). Few studies have investigated the relationship with acute infection of the SARS-CoV-2 virus and sleep and to our knowledge, all have been limited to only hospitalized patients (18, 19). However, non-hospitalized COVID-19 cases make up the majority of COVID-19 cases (1). Understanding how the pandemic, as well as SARS-CoV-2 infection alters sleep habits among non-hospitalized individuals, is important to better identify consequential health outcomes of COVID-19.

The Arizona CoVHORT provides an opportunity to evaluate the association of COVID-19 illness and sleep in a cohort of Arizona residents with laboratory confirmed SARS-CoV-2 who were not hospitalized for their COVID-19 illness. The main objectives of this analysis were to (1) describe sleep habits (sleep duration, time to bed, time awake, sleep disturbance) of all participants before and during the COVID-19 pandemic; (2) compare the sleep duration and sleep disturbance during the pandemic of SARS-CoV-2 positive compared to SARS-CoV-2 negative participants (3) assess change in sleep duration from prior to the pandemic compared to during the pandemic among SARS-CoV-2 positive and SARS-CoV-2 negative participants. We also investigated whether there were differences among SARS-CoV-2 positive participants by symptom prevalence (symptomatic and asymptomatic) and timing of questionnaire (infection <30 days prior to questionnaire and >30 days before questionnaire completion) compared to SARS-CoV-2 negative participants.

## Materials and Methods

### Study Design

The Arizona CoVHORT is a prospective cohort of Arizona residents with and without suspected and confirmed SARS-CoV-2 infection since May 2020. The overarching goal of the CoVHORT is to identify COVID-19 risk factors and subsequent chronic health conditions post-infection and describe associated symptoms and disease severity. As described previously (20), the cohort was launched on May 28, 2020 and has recruited over 6,400 participants as of August 2021. Baseline data are collected using a 95-item online questionnaire. All participants of the cohort receive quarterly follow-up questionnaires at 3, 6, 9, and 12 months. Individuals who report a laboratory-confirmed COVID-19 illness receive additional follow-up questionnaires that are distributed 6 weeks following the survey at which they report their positive test.

Participants were recruited from three primary sources: case investigation follow-up, partnered research studies, and by postcards/flyers. Partnerships with the Arizona Department of Health Services, Arizona State University, and the State of Arizona COVID-19 Antibody Testing Initiative allowed individuals with laboratory-confirmed PCR, antigen and antibody test results (both positive and negative) to be contacted by email with information about the study, including links to the recruitment and consent materials. An additional sample of participants without SARS-CoV-2 infection were recruited through postcards and flyers. Informed consent was provided by all participants *via* online form. Ethical approval was obtained from the University of Arizona Institutional Review Board (Protocol #2003521636A002).

### COVID-19 Diagnosis

For this analysis, only participants with a laboratory-confirmed positive PCR or antigen test were considered “SARS-CoV-2 positive.” Participants with negative test results and no prior positive test were considered “SARS-CoV-2 negative” and participants who had never completed a COVID-19 PCR or antigen test were considered “SARS-CoV-2 untested.”

### Sleep

For participants who enrolled prior to November 1, 2020, sleep questions were asked on the 3-month follow-up questionnaire. For participants who enrolled on or after November 1, 2020, sleep questions were asked at baseline. Participants were asked “What time did you usually go to bed before the COVID-19 pandemic?” “What time do you usually go to bed currently?” “What time did you usually wake up before the COVID-19 pandemic?” and “What time do you usually wake up currently?” Responses were used to calculate sleep duration pre-pandemic and during the pandemic. Based on other literature (21, 22), bedtime categories were created using reported time to bed (earlier than 10 pm = early; 10 pm to 12 am = mid; later than 12 am = late). Shift in bedtime was defined as the change in bedtime category from pre-pandemic to during the pandemic. To assess trouble sleeping, participants were asked “Since the start the COVID-19 pandemic, how often have you had trouble sleeping? This can be due to reasons such as trouble falling asleep, waking up early/in the middle of the night, trouble breathing/coughing/snoring, feeling too hot/cold, bad dreams, or pain” with response options of “not during the past month, less than once a week, once or twice a week, and three or more times a week.” We compressed these responses into two levels to investigate severe sleep disturbances: “less than three or more times a week” (reference category) and “three or more times a week.”

### Covariates

Baseline covariates were collected *via* self-reported online questionnaires and included age, gender, race, ethnicity, education, occupation, remote work status, income, body mass index (BMI), nicotine and marijuana smoking and vaping status, stress and emotional well-being construct score, and prevalent comorbid conditions. Gender categories included male, female, and non-binary or transgender male or female. Due to the small sample seen in the non-binary or transgender male or female category, only the male and female categories were included in the analyses. The Perceived Stress Scale (PSS)–10 was used to measure stress and emotional well-being with possible scores ranging from 0 to 40 where lower scores indicate more favorable health outcomes. SARS-CoV-2 positive participants were asked to report all symptoms they experienced during their illness, one of which was fatigue. The number of symptoms reported were then summed.

### Inclusion/Exclusion

For this analysis, we included participants who were 18 years or older who had complete exposure and outcome data collected by March 18, 2021. Participants who were pregnant (*n* = 44), had only reported a positive COVID-19 antibody test result (*n* = 64), or reported being hospitalized for their COVID-19 illness (*n* = 42) were excluded. Additionally, participants who tested negative for SARS-CoV-2 or who were untested were asked to report if they experienced any sudden, “COVID-19-like illness” since the start of the pandemic. If they answered yes, they were excluded from this analysis. To better assess the acute effects of COVID-19, we excluded participants whose test date was more than 30 days prior to the date of the baseline survey completion in the primary analyses. These participants were included in secondary analyses that investigated variability between time from infection to questionnaire completion.

## Statistical Methods

Descriptive statistics at baseline were stratified by SARS-CoV-2 test status (SARS-CoV-2 positive, SARS-CoV-2 negative, and SARS-CoV-2 untested). Sleep characteristics were stratified by SARS-CoV-2 test status. We used linear regression models to evaluate the difference in sleep duration of SARS-CoV-2 positive and SARS-CoV-2 negative participants. To investigate the difference in trouble sleeping between the SARS-CoV-2 positive and negative groups, we used logistic regression. We used linear mixed-effects models with a random effect to estimate the change in sleep duration from pre-pandemic to during the pandemic for SARS-CoV-2 positive and SARS-CoV-2 negative participants. The difference in sleep change between test groups was estimated from these mixed models. All regression models were adjusted for age, gender (male or female), and BMI categories (<18.5 kg/m^2^, 18.5–24.9 kg/m^2^, 25.0–29.9 kg/m^2^, and ≥30.0 kg/m^2^). To test if other potential confounders should be included, we added covariates (race, ethnicity, education, employment status, nicotine and marijuana smoking and vaping status, stress and emotional well-being construct score, and prevalent comorbid conditions) individually to the multivariable model. If the estimate changed by more than 10% the covariate was included in the model. None of the suspected cofounders met this criterion. A missing indicator was created for all included variables and frequencies are noted in the footnotes.

Two secondary analyses were completed to further investigate the difference in sleep duration and trouble sleeping among SARS-CoV-2 positive participants. First, we classified SARS-CoV-2 positive participants by symptom presentation during the acute phase of infection: SARS-CoV-2 positive participants who reported any symptoms, and SARS-CoV-2 positive participants who did not report symptoms (asymptomatic). Second, to see if time since infection influenced sleep outcomes, we completed the analyses comparing the following groups: diagnosis of SARS-CoV-2 ≤ 30 days prior to questionnaire completion, diagnosis of SARS-CoV-2 > 30 days from questionnaire completion, and SARS-CoV-2 negative (referent). The cut point of 30 days was established based on current CDC guidelines for post-COVID conditions, otherwise known as post-acute sequelae of SAR-CoV-2 (PASC) (23). A sensitivity analysis was also performed to expand the comparison group. In this analysis, individuals who tested negative and individuals who were untested were included in the reference category. All analyses were conducted using SAS 9.4 (SAS Institute, Cary, NC, USA).

## Results

A total of 1,848 CoVHORT study participants were included in our analysis. Overall, 16.2% were SARS-CoV-2 positive, 64.3% were SARS-CoV-2 negative, and 19.5% were untested for SARS-CoV-2 (Table 1). Generally, SARS-CoV-2 positive participants were younger, more likely to report obtaining less than a college degree, and more likely to report higher levels of perceived stress. Among the SARS-CoV-2 positive participants, 16.7% reported having an asymptomatic infection. Among the remaining 249 (83.3%) participants who experienced COVID-19 symptoms, fatigue was the most common symptom reported at baseline (64.9%).

**Table 1:** Characteristics of the Arizona CoVHORT study participants stratified by SARS-CoV-2 infection status, *n* = 1,848.

**Baseline characteristics**	**SARS-CoV-2 positive**	**SARS-CoV-2 negative**	**SARS-CoV-2 untested**
	**299 (16.2)**	**1,188 (64.3)**	**361 (19.5)**
	**Mean (SD)**		
Age, years	42.5 (17.2)	47.3 (15.8)	51.7 (15.8)
BMI, kg/m2	27.6 (7.4)	26.6 (6.3)	26.8 (5.7)
	***N*** **(%)**^**a**^		
**Gender**			
Male	83 (27.8)	384 (32.3)	95 (26.3)
Female	214 (71.6)	789 (66.4)	264 (73.1)
Non-binary or transgender male or female	2 (0.7)	12 (1.0)	2 (0.6)
**Race**			
American Indian or Alaskan Native	3 (1.0)	3 (0.3)	0 (0)
Asian	3 (1.0)	6 (0.5)	7 (1.9)
Black or African American	3 (1.0)	46 (3.9)	2 (0.6)
Native Hawaiian or Pacific Islander	0 (0)	15 (1.3)	0 (0)
Mixed Race	9 (3.0)	43 (3.6)	16 (4.4)
White	269 (90.0)	1,057 (89.0)	330 (91.4)
Prefer not to answer	12 (4.0)	17 (1.4)	2 (0.6)
Hispanic Ethnicity	62 (20.7)	131 (11.0)	39 (10.8)
**Education** ^**b**^			
Less than a college degree	108 (36.1)	182 (18.0)	24 (17.8)
**Employment status**			
Part- or full-time remote	111 (37.1)	515 (43.4)	147 (40.7)
Part- or full-time not remote	100 (33.4)	351 (29.5)	84 (23.3)
Other	88 (29.4)	322 (27.1)	130 (36.0)
**Income** ^**b**^			
<50 k	86 (28.8)	165 (16.4)	16 (11.9)
50–70 k	43 (14.4)	163 (16.2)	21 (15.6)
>70 k	134 (44.8)	575 (57.0)	69 (51.1)
Don't know / not sure or prefer not to answer	36 (12.0)	106 (10.5)	29 (21.5)
**BMI category, kg/m** ^**2**^			
Underweight (<18.5)	22 (7.4)	85 (7.2)	21 (5.8)
Normal weight (18.5–24.9)	102 (34.1)	486 (40.9)	135 (37.4)
Overweight (25.0–29.9)	83 (27.8)	354 (29.8)	127 (35.2)
Obese (≥30.0)	91 (30.4)	254 (21.4)	77 (21.3)
Ever smoke or vape nicotine	29 (9.7)	83 (7.0)	15 (4.2)
Smoke or vape marijuana occasionally or regularly	35 (11.7)	132 (11.1)	36 (10.0)
**Stress and emotional wellness, construct score** ^**c**^			
Low stress (0–13)	81 (27.1)	475 (40.0)	189 (52.4)
Moderate stress (14–26)	185 (61.9)	605 (50.9)	151 (41.8)
High perceived stress (27–40)	33 (11.0)	105 (8.8)	21 (5.8)
**Prevalent comorbidities**			
Hypertension	53 (17.7)	198 (16.7)	73 (20.2)
CVD^d^	17 (5.7)	45 (3.8)	16 (4.4)
Diabetes	12 (4.0)	36 (3.0)	21 (5.8)
Depression / Anxiety	30 (10.0)	126 (10.6)	41 (11.4)
**Reported symptoms**			
None	50 (16.7)		
1–6	114 (38.1)		
7–12	123 (41.1)		
13+	12 (4.0)		
Symptom^e^–fatigue	194 (64.9)		

*BMI, body mass index; CVD, cardiovascular disease*.

a *Categories may not sum to 100% due to missing data: race, n = 7; ethnicity, n = 26; BMI, n = 11; smoke or vape nicotine, n = 24; smoke or vape marijuana, n = 25; stress and emotional wellness, n = 3*.

b *Does not reflect first 405 participants enrolled in study*.

c *Scored using the Perceived Stress Scale (PSS) – 10 with possible scores ranging from 0 to 40 where lower scores indicate more favorable health status*.

d *Includes self-reported history of stroke, myocardial infarction, congestive heart failure, angina, and other cardiovascular disease*.

e *Possible symptoms include fever, sore throat, cough, difficulty breathing or shortness of breath, chest pain or pressure, runny nose/cold-like symptoms, fatigue, aches and pains or sore muscles, chills, diarrhea, nausea, vomiting, headache, loss of smell/taste, bone pain/nerve pain, conjunctivitis, rash on skin, discoloration of fingers and toes, loss of speech or movement, and other*.

When comparing sleep duration prior to and during the pandemic, average sleep duration for both SARS-CoV-2 positive and SARS-CoV-2 negative participants increased during the pandemic compared to prior to the pandemic (Table 2). However, among SARS-CoV-2 positive participants, sleep duration increased by more than 1 h (Prior: 8.1 h, During: 9.4). SARS-CoV-2 positive participants had the highest probability of having trouble sleeping three of more times per week (35.5%) compared to SARS-CoV-2 negative (28.8%) and untested participants (28.5%). Among the COVID-19 positive participants, 23.4% shifted to an earlier bedtime, which is nearly double what was observed among SARS-CoV-2 negative (12.2%) and SARS-CoV-2 untested (10.2%) participants.

**Table 2:** Cross-sectional sleep characteristics of participants in the Arizona CoVHORT Study, prior to the COVID-19 pandemic and during the pandemic, *n* = 1,848.

**Sleep measurements mean (SD) or *n* (%)**	**SARS-CoV-2 positive**	**SARS-CoV-2 negative**	**SARS-CoV-2 untested**
	**299 (16.2)**	**1,188 (64.3)**	**361 (19.5)**
**Time to bed, hh:mm**			
Before the pandemic	22:24 (1:19)	22:12 (1:25)	22:05 (1:19)
Currently	22:17 (1:52)	22:17 (1:38)	22:11 (1:30)
**Time awake, hh:mm**			
Before the pandemic	6:28 (1:36)	6:18 (1:18)	6:12 (1:21)
Currently	7:38 (2:01)	6:37 (1:34)	6:22 (1:27)
**Sleep duration, hours**			
Before the pandemic	8.1 (1.3)	8.1 (1.3)	8.1 (1.2)
Currently	9.4 (2.0)	8.3 (1.4)	8.2 (1.2)
**Trouble sleeping since the start of the pandemic**			
Not during the past month	61 (20.4)	236 (19.9)	79 (21.9)
Less than once a week	51 (17.1)	266 (22.4)	68 (18.8)
Once or twice a week	81 (27.1)	344 (29.0)	111 (30.7)
Three or more times a week	106 (35.5)	342 (28.8)	103 (28.5)
**Bedtime before the pandemic**			
Early (before 10 pm)	130 (43.5)	531 (44.7)	171 (47.4)
Mid (10 pm−12 am)	122 (40.8)	553 (46.5)	162 (44.9)
Late (after 12 am)	47 (15.7)	104 (8.8)	28 (7.8)
**Bedtime currently**			
Early (before 10 pm)	141 (47.2)	537 (45.2)	171 (47.4)
Mid (10 pm−12 am)	108 (36.1)	507 (42.7)	148 (41.0)
Late (after 12 am)	50 (16.7)	144 (12.1)	42 (11.6)
**Shift in bedtime**			
No shift	173 (57.9)	869 (73.1)	275 (76.2)
Shifted later	56 (18.7)	174 (14.6)	49 (13.6)
Shifted earlier	70 (23.4)	145 (12.2)	37 (10.2)

*h, hour; m, minute, SD, standard deviation*.

During the pandemic, laboratory-confirmed SARS-CoV-2 positive participants reported over 60 minutes longer of sleep duration than SARS-CoV-2 negative participants (difference in duration in minutes: 60.9, 95% CI 49.1, 72.8) (Table 3). SARS-CoV-2 positive participants had greater odds of having more trouble sleeping (OR: 1.34 95% CI 1.02, 1.77) compared to SARS-CoV-2 negative participants. There was a statistically significant increase in sleep duration from pre-pandemic compared to during the pandemic for both SARS-CoV-2 positive (change in duration: 77.7 min, 95% CI 67.9, 87.5) and SARS-CoV-2 negative participants (change in duration: 13.4 min, 95% CI 8.4, 18.3) (Table 4). The increase for SARS-CoV-2 positive participants from pre-pandemic to during the pandemic was higher than for negative participants by 64.3 min (95% CI 53.3, 75.3; *p* < 0.001). Results remained similar in sensitivity analyses including individuals untested for SARS-CoV-2 in the referent category (Supplementary Table 1).

**Table 3:** Difference in sleep duration and trouble sleeping during the COVID-19 pandemic among participants in the Arizona CoVHORT Study by test group, presentation of symptoms, and time since SARS-CoV-2 test, *n* = 1,487.

		**Unadjusted**	**Adjusted^**a**^**
**Sleep Duration**			
	**Mean (SD), hours**	**Estimated sleep duration (95% CI), minutes**	
**Overall**			
SARS-CoV-2 negative	8.3 (1.4)	0.0 (Ref)	0.0 (Ref)
SARS-CoV-2 positive	9.4 (2.0)	63.0 (51.2, 74.8)	60.9 (49.1, 72.8)
**Presentation of symptoms**			
SARS-CoV-2 negative	8.3 (1.4)	0.0 (Ref)	0.0 (Ref)
SARS-CoV-2 positive symptomatic	9.5 (2.0)	70.8 (58.2, 83.5)	68.8 (56.1, 81.5)
SARS-CoV-2 positive asymptomatic	8.7 (1.5)	23.9 (-2.2, 50.1)	22.1 (-3.9, 48.2)
**Time since SARS-CoV-2 test** ^**b**^			
SARS-CoV-2 negative	8.3 (1.4)	0.0 (Ref)	0.0 (Ref)
SARS-CoV-2 positive ≤ 30 days	9.4 (2.0)	63.0 (51.1, 74.9)	61.3 (49.3, 73.4)
SARS-CoV-2 positive > 30 days	8.9 (1.8)	35.8 (19.5, 52.1)	35.2 (18.8, 51.7)
**Trouble sleeping ≥3 times per week**			
	***N*** **(%)**	**OR (95% CI)**	**OR (95% CI)**
**Overall**			
SARS-CoV-2 negative	342 (28.8)	1.00 (Ref)	1.00 (Ref)
SARS-CoV-2 positive	106 (35.5)	1.36 (1.04, 1.78)	1.34 (1.02, 1.77)
**Presentation of symptoms**			
SARS-CoV-2 negative	342 (28.8)	1.00 (Ref)	1.00 (Ref)
SARS-CoV-2 positive symptomatic	87 (34.9)	1.33 (1.00, 1.77)	1.31 (0.97, 1.76)
SARS-CoV-2 positive asymptomatic	19 (38.0)	1.52 (0.85, 2.72)	1.55 (0.86, 2.80)
**Time since SARS-CoV-2 test**			
SARS-CoV-2 negative	342 (28.8)	1.00 (Ref)	1.00 (Ref)
SARS-CoV-2 positive ≤ 30 days	106 (35.5)	1.36 (1.04, 1.78)	1.34 (1.02, 1.77)
SARS-CoV-2 positive > 30 days	66 (46.2)	2.12 (1.49, 3.02)	2.11 (1.47, 3.03)

*SD, standard deviation; CI, confidence interval; OR, odds ratio*.

a *Adjusted for age, gender, and BMI and missing covariates are as follows: gender, n = 17; BMI, n = 10*.

b *N = 1,630; missing covariates in adjusted models are as follows: gender, n = 18; BMI, n = 12*.

**Table 4:** Change in sleep duration among CoVHORT study participants from pre-pandemic to during the pandemic by SARS-CoV-2 test group, presentation of symptoms, and time since SARS-CoV-2 test, *n* = 1,487.

	**Unadjusted**	**Adjusted^**a**^**
	**Estimated change in duration of sleep prior to the pandemic compared to during the pandemic (95% CI), minutes**	
**Overall**		
SARS-CoV-2 negative	13.5 (8.6, 18.5)	13.4 (8.4, 18.3)
SARS-CoV-2 positive	77.2 (67.4, 87.1)	77.7 (67.9, 87.5)
**Presentation of symptoms**		
SARS-CoV-2 negative	13.5 (8.6, 18.4)	13.4 (8.5, 18.3)
SARS-CoV-2 positive symptomatic	86.1 (75.3, 96.8)	86.8 (76.1, 97.5)
SARS-CoV-2 positive asymptomatic	33.0 (9.0, 57.0)	33.0 (9.2, 56.8)
**Time since SARS-CoV-2 test** ^**b**^		
SARS-CoV-2 negative	13.5 (8.5, 18.6)	13.4 (8.3, 18.5)
SARS-CoV-2 positive ≤ 30 days	77.2 (67.2, 87.3)	77.7 (67.6, 87.7)
SARS-CoV-2 positive > 30 days	45.7 (31.2, 60.3)	46.2 (31.5, 60.8)

*CI, confidence interval*.

a *Adjusted for age, gender, and BMI and missing covariates are as follows: gender, n = 17; BMI, n = 10*.

b *N = 1,630; missing covariates in adjusted models are as follows: gender, n = 18; BMI, n = 12*.

When investigating differences by symptom presentation among SARS-CoV-2 positive participants, symptomatic SARS-CoV-2 positive participants slept 68.8 min (95% CI 56.1, 81.5), while asymptomatic SARS-CoV-2 positive participants slept 22.1 min longer (95% CI −3.9, 48.2) compared to SARS-CoV-2 negative participants (Table 3). Trouble sleeping was elevated among both symptomatic and asymptomatic SARS-CoV-2 positive individuals, however, was not statistically significant among either group (symptomatic OR: 1.31 95% CI 0.97, 1.76; asymptomatic OR: 1.55 95% CI 0.86, 2.80), possibly due to limited statistical power. Results from the mixed models showed that symptomatic SARS-CoV-2 positive participants slept 86.8 min (95% CI 76.1, 97.5) longer while asymptomatic SARS-CoV-2 positive participants slept 33.0 min (95% CI 9.2, 56.8) longer after infection compared to pre-pandemic (Table 4).

We observed that differences in sleep between SARS-CoV-2 positive and negative participants persisted more than 30 days after infection. In secondary analyses where we included SARS-CoV-2 positive individuals who completed the sleep questionnaire > 30 days after receiving a positive SARS-CoV-2 test result, we observed that SARS-CoV-2 positive participants slept 35.2 min longer (95% CI 18.8, 51.7) and experienced greater odds of trouble sleeping (OR: 2.11 95% CI 1.47, 3.03) compared to SARS-CoV-2 negative participants (Table 3). These individuals also reported sleeping 46.2 min longer (95% CI 31.5, 60.8) than they did before the pandemic (Table 4).

## Discussion

Results from this cohort study of Arizona residents suggests that individuals with a laboratory-confirmed SARS-CoV-2 infection slept longer than individuals without SARS-CoV-2 infection and that SARS-CoV-2 infection increased odds of trouble sleeping. Compared to sleep duration pre-pandemic, both SARS-CoV-2 positive and SARS-CoV-2 negative participants slept longer during the pandemic; however, the change in sleep duration was greater among positive participants than negative participants. Further, our findings suggest that (1) the presence of symptoms during COVID-19 illness influenced sleep behaviors and (2) COVID-19 illness may be contributing to long-term sleep changes observed more than 30 days following a positive SARS-CoV-2 test result.

Prior to the pandemic, about one-third of adults in the U.S. were not getting adequate sleep per night, defined as 7–9 h (24). Results from our analysis show that sleep duration increased during the pandemic for all participants, regardless of SARS-CoV-2 infection status. These results indicate that the pandemic, not just SARS-CoV-2 infection, has an impact on sleep habits, which is consistent with other studies (10, 25, 26). Later average wake-up time observed during the pandemic for all test groups may have contributed to longer sleep durations. This may be the result of more relaxed or lack of rigid morning start times due to remote work or school. Change in morning start time may also explain the shift in bedtime seen among ~36% of our study participants. Almost 17% of study participants shifted to an earlier bedtime which differs from results of previous studies that have reported significant delays in bedtime (12, 27). These findings may suggest that the pandemic gave individuals the opportunity to adhere to their natural sleep-wake cycle instead of matching sleep-wake habits to meet the demands of work and school schedules.

The increased sleep duration and altered sleep timing seen among SARS-CoV-2 positive participants may be the result of SARS-CoV-2 infection. There is strong evidence of the bidirectional association of altered sleep and infection (6). These associations vary depending on the pathogen (14) and have not been well-explored with the SARS-CoV-2 virus. Generally, acute infectious illnesses induce a sickness behavior characterized by fever, fatigue, pain hypersensitivity, loss of appetite, and altered sleep (28). Sickness behavior is mediated by cytokines (28), which are also known to influence sleep (29). Sleep duration and timing among SARS-CoV-2 positive participants may have been influenced by sickness behavior as these participants reported current sleep within 30-days of their SARS-CoV-2 test. Altered sleep among SARS-CoV-2 positive individuals may also have been impacted by self-isolation or time off of work due to infection status.

Among individuals who tested positive for SARS-CoV-2 and experienced symptoms (84%), fatigue was the most commonly reported symptom with 65% of participants reported experiencing fatigue within 30 days of their positive test result. Further, a previous study by Bell et al. reported the presence of post-acute sequelae of SAR-CoV-2 (PASC) among 68.7% of SARS-CoV-2 positive CoVHORT study participants with ≥30 days of follow-up as of February 24, 2021 (30). PASC, often known as “long COVID,” is a loosely defined term to describe when symptoms of COVID-19 illness persist beyond the initial infectious period (30). Among these individuals, 37.5% reported that they were still experiencing fatigue. Generally, sleep quality is thought to be associated with fatigue where poor perceived sleep quality leads to increased levels of fatigue (31). While we observed that SARS-CoV-2 positive participants who reported fatigue as a symptom had increased trouble sleeping, we did not investigate the potential bidirectional relationship of this association. Further research is needed to better understand the relationship between the presence of fatigue due to SARS-CoV-2 infection and PASC with sleep.

Previous studies have shown that sleep quality among hospitalized COVID-19 patients is poor (18). Results from our current study show that non-hospitalized individuals with COVID-19 also report more trouble sleeping compared to SARS-CoV-2 negative participants. Interestingly, individuals who reported sleep habits more than 30 days after their positive SARS-CoV-2 test had higher odds (OR: 2.11) of trouble sleeping than individuals who reported sleep habits within 30 days of their positive SARS-CoV-2 test (1.34). This suggests that sleep quality may continue to worsen even after acute SARS-CoV-2 infection. A previous study of hospitalized COVID-19 patients reported sleep difficulties among 25% of patients with PASC (32). Yet, little is known about how PASC may influence sleep habits among non-hospitalized individuals with COVID-19. Further research is needed to understand the potential relationship between the long-term effects of COVID-19 illness and sleep after acute infection with the SARS-CoV-2 virus for individuals with and without PASC.

Frequency of trouble sleeping prior to the pandemic was not collected so we were unable to determine if sleep difficulties increased during the pandemic despite SARS-CoV-2 infection. However, the challenges brought on by the pandemic such as disrupted schedules, increased stress, increased screen-time, disruption in physical activity and dietary habits, and increased feelings of isolation are known to influence sleep quality. Although we were unable to explore these factors as they related to sleep difficulties in this study, we suspect these factors may have influenced sleep in our study population. Previous studies suggest these environmental, social, and lifestyle factors indeed disrupted sleep habits among the general population during the COVID-19 pandemic (13, 33). The presence of routine sleep disturbances may have major public health implications as sleep quality is closely related to cognitive and mental health (34) which have been challenged for many during the pandemic (35).

This analysis has several strengths including its large sample size and recruitment from the general population allowing for increased generalizability to US residents. Additionally, the inclusion of non-hospitalized individuals with laboratory-confirmed SARS-CoV-2 infection presents a unique population, generalizable to the majority of COVID-19 cases, as all prior studies have been restricted to hospitalized patients (18, 19, 36). While the current study provides important advancements in our understanding of how sleep habits have changed due to SARS-CoV-2 infection and the COVID-19 pandemic, there are limitations to consider. The sample was predominantly white race which reduces the representativeness and limits generalizability. Sleep was collected using self-reported questionnaires rather than validated questionnaires or objective measurement devices. Psychological and cognitive arousal (i.e., stress, worry, and/or anxiety) can result in worse subjective reporting of sleep quantity and quality (37). Such arousal is reported to be associated with the unprecedented challenges brought on by the pandemic (35), and therefore may influence the subjective measurements of sleep reported in this study. There was variation in questionnaires, between May 2020 and November 2020, sleep was collected at 3-month follow-up and after November 2020 sleep was collected at baseline. Due to limited access to SARS-CoV-2 testing at the start of the pandemic, some individuals did not receive confirmed laboratory testing but did report having COVID-19-like symptoms; however, these participants were excluded from analyses. Lastly, sedentary time and physical activity were not collected and therefore were not assessed as potential confounders.

Our findings suggest that during the COVID-19 pandemic sleep duration increased regardless of SARS-CoV-2 infection. Sleep duration and trouble sleeping among SARS-CoV-2 positive individuals varied based on time since infection and presentation of symptoms. Individuals with a positive SARS-CoV-2 test result were more likely to report trouble sleeping three or more times per week since the start of the pandemic. Overall, sleep habits have changed because of SARS-CoV-2 infection and the COVID-19 pandemic. Although the shift toward longer sleep duration could be beneficial for some, longer sleep durations can potentially have negative health consequences. The persistence of sleep difficulties among SARS-CoV-2 positive individuals more than 30 days after a positive test result may lead to additional adverse health effects and should be of concern among public health professionals. Longer term follow-up is needed to determine if sleep habits continue to evolve post SARS-CoV-2 infection and as stay-at-home orders are lifted and many individuals return to in-person work and school commitments.

## Data Availability Statement

Due to privacy and ethical considerations, data are not publicly available.

## Ethics Statement

The studies involving human participants were reviewed and approved by University of Arizona Institutional Review Board (Protocol #2003521636A002). The patients/participants provided their written informed consent to participate in this study.

## Author Contributions

SMD: statistical analysis plan, data analysis, interpretation of results, and writing and editing of the manuscript. LK, EJ, KP-B, and KE: conceptualization and editing the manuscript. TC: statistical analysis plan, conceptualization, interpretation of results, and editing the manuscript. MB: data analysis, interpretation of results, and editing the manuscript. CC and RB: data collection and editing the manuscript. LF: statistical analysis plan, conceptualization, interpretation of results, supervision, and editing of the manuscript. All authors contributed to the article and approved the submitted version.

## Funding

This study has been funded by a seed grant awarded to KP-B and EJ by the BIO5 Institute at the University of Arizona. The sponsors have no role or authority over the design or implementation of this study.

## Conflict of Interest

The authors declare that the research was conducted in the absence of any commercial or financial relationships that could be construed as a potential conflict of interest.

## Publisher's Note

All claims expressed in this article are solely those of the authors and do not necessarily represent those of their affiliated organizations, or those of the publisher, the editors and the reviewers. Any product that may be evaluated in this article, or claim that may be made by its manufacturer, is not guaranteed or endorsed by the publisher.
