# Supplementary material for: COVID-19 Infection, the COVID-19 Pandemic, and Changes in Sleep
Source: Front Public Health. 2022 Jan 31;9:795320. doi: 10.3389/fpubh.2021.795320 (PMC8841649; doi:10.3389/fpubh.2021.795320)
Supplement: Supplementary file 1 [file Table_1.docx]

**Title:** COVID-19 infection, the COVID-19 pandemic, and changes in sleep

**Authors**: Sidney M. Donzella^1,2^, Lindsay N. Kohler^1,3,4^, Tracy E. Crane^5^, Elizabeth T. Jacobs^1,3^, Kacey C. Ernst^1^, Melanie L. Bell^1^, Collin J. Catalfamo^1^, Rachelle Begay^1^, Kristen Pogreba-Brown^1^, Leslie V. Farland^1^

**Affiliations**

^1^ Department of Epidemiology and Biostatistics, University of Arizona

^2^ Department of Epidemiology, University of Washington

^3^ University of Arizona Cancer Center

^4^ Department of Health Promotion Science, University of Arizona

^5^ Department of Medicinal Oncology, University of Miami

**Corresponding author**:

Sidney Donzella

sdonz@uw.edu

**Supplemental Table 1**. Difference in sleep duration and trouble sleeping during the COVID-19 pandemic among participants in the Arizona CoVHORT Study by test group with untested participants in the reference group in sensitivity analyses

|  | Primary Adjusted^a^ | | Sensitivity Adjusted^ab^ | |
| --- | --- | --- | --- | --- |
|  | n=1,487 | | n=1,848 | |
| **Sleep Duration** |  |  |  |  |
|  | Mean (SD), hours | Estimated sleep duration (95% CI), minutes | Mean (SD), hours | Estimated sleep duration (95% CI), minutes |
| SARS-CoV-2 negative | 8.3 (1.4) | Reference | 8.3 (1.4) | Reference |
| SARS-CoV-2 positive | 9.4 (2.0) | 60.9 (49.1, 72.8) | 9.4 (2.0) | 63.3 (52.1, 74.4) |
| **Trouble sleeping ≥ 3 times per week** | |  |  |  |
|  | N (%) | OR (95% CI) | N (%) | OR (95% CI) |
| SARS-CoV-2 negative | 342 (28.8) | Reference | 445 (28.7) | Reference |
| SARS-CoV-2 positive | 106 (35.5) | 1.34 (1.02, 1.77) | 106 (35.5) | 1.36 (1.04, 1.77) |

Abbreviations: CI, confidence interval; OR, odds ratio

^a^ Adjusted for age, gender, and BMI and missing covariates are as follows: gender, n=17; BMI, n=10

^b^ SARS-CoV-2 category includes participant with confirmed COVID-19 negative test results and untested participants; missing covariates in adjusted models are as followed: gender, n=19; BMI, n=11

**Supplemental Table 2.** Comparison of primary and sensitivity analyses for Change in sleep duration from pre-pandemic to during the pandemic by COVID-19 test group, presentation of symptoms, and time since COVID-19 test

|  | Primary Adjusted^a^ | Sensitivity Adjusted^ab^ |
| --- | --- | --- |
|  | Estimated change in duration (95% CI), minutes | Estimated change in duration (95% CI), minutes |
|  | n=1,484 | n=1,848 |
| SARS-CoV-2 negative | 13.4 (8.4, 18.3) | 11.0 (6.9, 15.1) |
| SARS-CoV-2 positive | 77.7 (67.9, 87.5) | 77.7 (68.4, 87.0) |

^a^ Adjusted for age, gender, and BMI and missing covariates are as follows: gender, n=17; BMI, n=10

^b^ SARS-CoV-2 category includes participant with confirmed COVID-19 negative test results and untested participants; missing covariates in adjusted models are as followed: gender, n=19; BMI, n=11
